# Direct comparison of two different mesalamine formulations for the maintenance of remission in patients with ulcerative colitis: A double-blind, randomized study

**DOI:** 10.1002/ibd.21194

**Published:** 2010-01-04

**Authors:** Hiroaki Ito, Mitsuo Iida, Takayuki Matsumoto, Yasuo Suzuki, Yoshiyuki Aida, Toyomitsu Yoshida, Yuichi Takano, Toshifumi Hibi

**Affiliations:** * Digestive Disease Center of Excellence, Kitano Hospital, The Tazuke Kofukai Medical Research InstituteOsaka, Japan; † Department of Medicine and Clinical Science, Graduate School of Medical Science, Kyushu UniversityFukuoka, Japan; ‡ Division of Lower Gastroenterology, Department of Internal Medicine, Hyogo College of MedicineHyogo, Japan; § Department of Internal Medicine, Toho University Sakura Medical CenterChiba, Japan; ∥ Clinical Research, ZERIA Pharmaceutical Co., Ltd.Tokyo, Japan; ¶ Division of Gastroenterology and Hepatology, Department of Internal Medicine, Keio University School of MedicineTokyo, Japan

**Keywords:** mesalamine, ulcerative colitis, randomized controlled trial, colonoscopy

## Abstract

**Background:**

Mesalamine has been used as the first-line medication for the treatment of ulcerative colitis (UC). We directly compared the efficacy and safety of two different mesalamine formulations in the maintenance of remission in patients with UC.

**Methods:**

In a multicenter, double-blind, randomized study, 131 patients with quiescent UC were assigned to two groups: 65 to receive a pH-dependent release formulation of mesalamine at 2.4 g/day (pH-2.4 g) and 66 to receive a time-dependent release formulation of mesalamine at 2.25 g/day (Time-2.25 g). Both formulations were administered three times daily for 48 weeks. The primary endpoint was the proportion of patients without bloody stools.

**Results:**

In the full analysis set (*n* = 130), the proportion of patients without bloody stools was 76.9% in the pH-2.4 g and 69.2% in the Time-2.25 g, demonstrating the noninferiority of pH-2.4 g to Time-2.25 g. No statistically significant difference in time to bloody stools was found between the two formulations (*P* = 0.27, log-rank test), but the time to bloody stools tended to be longer in pH-2.4 g compared to Time-2.25 g, and a similar trend was observed with regard to the time to relapse. No differences were observed between the safety profiles of the two formulations.

**Conclusions:**

The pH- and time-dependent release of mesalamine formulations were similarly safe and effective. Interestingly, the remission phase tended to be longer in the group that received the pH-dependent formulation compared to the group that received the time-dependent formulation (UMIN Clinical Trials Registry, no. C000000289). (Inflamm Bowel Dis 2010)

Ulcerative colitis (UC) is a chronic disease characterized by inflamed mucosa limited to the large intestine. The etiology of UC has not yet been elucidated. The major therapeutic approaches to UC include drug therapy and surgery, and the goal is long-term control of the disease condition in order to improve the patients' quality of life (QOL).^1^,^2^ Various formulations of mesalamine, corticosteroids and immunosuppressants have been utilized to treat UC. However, long-term treatment with oral mesalamine has been the key approach to the treatment of patients with UC, especially in the remission phase, because of its safety, effectiveness and its potential for a decrease in the risk of colorectal cancer.^3^,^4^

Because mesalamine exerts its effect directly on the inflamed mucosa, higher concentration of mesalamine in the mucosa is required to attain a more pronounced effect. However, since mesalamine is absorbed in the upper gastrointestinal tract,^5^–^7^ many controlled-release formulations of oral mesalamine, such as pH-dependent release and time-dependent release formulations, have been developed to enhance its effect. A time-dependent release formulation coated with ethyl cellulose (Pentasa) gradually releases mesalamine starting in the stomach,^7^,^8^ whereas a pH-dependent release formulation coated with Eudragit-S (Asacol) releases mesalamine in the distal ileum or colon, since the coating dissolves at pH 7 or higher.^7^–^9^

There has been no comparative study to assess the clinical effects of the two formulations with different release profiles, and thus little scientific evidence for choosing one formulation over the other is currently available for appropriate treatment. Therefore, we conducted a double-blind, randomized, controlled study aimed to clarify this issue, comparing the efficacy and safety of two types of release formulations in patients with UC in the remission- phase (UMIN Clinical Trials Registry, no. C000000289).

## MATERIALS AND METHODS

### Patient Selection

We conducted the study in patients with quiescent UC on the basis of two inclusion criteria: 1) outpatients who were 16–64 years of age at the time of the informed consent, and 2) patients who had quiescent UC defined by an UC disease activity index (UC-DAI) of 2 or less and a bloody stool score of 0. The UC-DAI was originally developed by Sutherland et al.^10^

The patients were excluded according to following criteria: 1) corticosteroids (oral preparations, enemas, suppositories, injections and/or remedies for hemorrhoidal diseases) and/or cytapheresis within 14 days before the start of the investigational drugs; 2) immunosuppressants within 90 days before the start of the investigational drug; 3) any other investigational drugs within six months before informed consent (except the investigational drugs in a study for active UC, UMIN Clinical Trials Registry, no. C000000288); 4) a history of hypersensitivity to mesalamine or salicylate drugs, severe cardiac disease, pulmonary disease and/or hematological disease; 5) severe hepatopathy, severe nephropathy and/or a malignant tumors; and 6) pregnant or lactating.

### Ethical Considerations

This study was conducted according to the principles of the Declaration of Helsinki after obtaining approvals from the Institutional Review Board at each of the participating medical centers. Written informed consent was obtained from all participants.

### Study Drugs

The pH-dependent release mesalamine formulation used in this study was a tablet coated with Eudragit-S (Asacol 400 mg tablet, Tillotts Pharma - AG, Ziefen, Switzerland, supplied by ZERIA Pharmaceutical, Tokyo, Japan). The time-dependent release mesalamine formulation used in this study was a tablet coated with ethyl cellulose (Pentasa 250 mg tablet, Nissin Kyorin Pharmaceutical, Japan). This study was conducted using a double-dummy method.

### Study Design

This double-blind, randomized, controlled study was conducted at 50 centers in Japan. Treatment assignments were balanced according to two patient demographics with the use of a biased-coin minimization algorithm. The first was prior participation in a study of the same two mesalamine formulations in patients with active UC conducted during the same period as the present study: UMIN Clinical Trials Registry, no. C000000288 (Yes or No). The second was the duration of the remission phase of UC (<2 years or ≥2 years). Balance within each medical center was also taken into consideration. A person independent from the study was in charge of the random allocation. Four patients were assigned as a block as follows: 2 to a group given the pH-dependent release mesalamine formulation at 2.4 g/day (pH-2.4 g) and 2 to a group given the time-dependent release mesalamine formulation at 2.25 g/day (Time-2.25 g). The randomization code was sealed and stored until the blind was removed.

At time of the informed consent, investigators evaluated the background characteristics of patients. After an observation period of 3–14 days from the time of informed consent, investigators assessed patients for their eligibility for enrolment according to criteria previously described. At the assessment for eligibility, the UC-DAI was calculated using a previously reported method.^11^,^12^ The UC-DAI is the sum of the mucosal appearance score (based on the colonoscopy findings by reference to atlases of mucol appearance), stool frequency score bloody stool score, and physician's global assessment score (stage 0, 1, 2, or 3). Each score was based on the patients' diary for the last three days. The area of the inflammation was also determined by colonoscopy. Patients who were judged as eligible were enrolled and assigned to investigational drugs by a central registration center, and then administration was started. The investigational drugs were administered three times daily for 48 weeks.

During the study, each patient recorded the condition of their bloody stools, stool frequency and drug compliance in their diary and visited the medical center every four weeks. Each component of UC-DAI, except the mucosal appearance score, was assessed at each visit.^11^,^12^ Colonoscopy was performed at 48 weeks or at withdrawal from the study, and the mucosal appearance score at that time was used to calculate UC-DAI. To evaluate safety, clinical laboratory data and vital signs were checked at the time of informed consent and every 12 weeks after enrolment (or upon withdrawal). The presence or absence of adverse events (AEs) and adverse drug reactions (ADRs) were recorded by investigators at each visit.

### Statistical Analysis

The primary endpoint was the proportion of patients without bloody stools. The presence of bloody stools was defined as a bloody stool score of 1 or more. The principal hypothesis was the noninferiority of pH-2.4 g to Time-2.25 g, using the proportion of patients without bloody stools.

Our hypothesis was verified by the following methods. The noninferiority of pH-2.4 g to Time-2.25 g was demonstrated if the lower limit of the 95% confidence interval (CI) was more than “−10.0%” in the difference of the proportion of patients without bloody stools between the two groups (pH-2.4 g minus Time-2.25 g). In addition, the superiority of pH-2.4 g over Time-2.25 g was demonstrated if the lower limit of the 95% CI was more than “0.0%” in the difference of the proportion of patients without bloody stools between the two groups. The secondary endpoints were time to bloody stools, proportion of patients without relapse, time to relapse and decrease in UC-DAI. In this study, relapse was defined as a bloody stool score of 1 or more and UC-DAI of 3 or more. Survival values of the time to events were determined by the Kaplan–Meier method with time to bloody stools and time to relapse. The functions were compared by the log-rank test and the hazard ratio (HR) of pH-2.4 g to Time-2.25 g and the 95% CI were calculated. A statistically significant difference was demonstrated when the 95% CI of the difference between the groups did not include zero with regard to proportions of patients without relapse and the decrease in UC-DAI. In the safety endpoints, the numbers of patients with AEs and patients with ADRs were analyzed by Fisher's exact test.

Unless otherwise specified, differences at α = 0.05 (two-sided) and *P* < 0.05 were considered statistically significant. The statistical analyses were conducted by ZERIA Pharmaceutical, Japan, based on statistical advice of an expert independent of this study.

The number of patients required to determine the hypotheses was estimated to be 57 at α = 0.05 (two-sided), β = 0.1 and Δ = 10% when the proportion of patients without bloody stool was 65% in pH-2.4 g and 45% in Time-2.25 g, respectively. According to the above estimations, we decided to enroll at least 60 patients in each group considering the patients excluded from the analysis set.

The full analysis set (FAS) consisted of all participants except those who had not taken even one tablet of the investigational drug, those who did not comply with Good Clinical Practice (GCP) and those whose data were missing at the efficacy endpoint. The per protocol set (PPS) consisted of the FAS except those who did not fulfill the inclusion criteria, those who met the exclusion criteria, those who received forbidden drugs and those whose drug compliance was less than 75%. Concerning the withdrawal cases, their adoption was to be decided before the bind was removed. The statistical analysis of efficacy was performed primarily based on data from the FAS followed by comparison with those in the PPS. The dataset for safety consisted of all participants except those who had not taken even one tablet of the investigational drug and those who did not comply with GCP.

### Independent Image Assessment Committee

We established an image assessment committee independent from the investigators to ensure the reliability of the mucosal appearance scores, and each of the three members of the committee blindly and independently scored the mucosal appearance by examining photos provided by the investigators. When the score obtained from all three members was the same, that score was regarded as a judgment by the committee. If the scores were different, the committee members discussed the case until they reached a consensus. When the judgment by the committee and the evaluation by the investigators were the same, it was defined as an agreement case.

## RESULTS

### Patient Demographics

**FIGURE 1:**
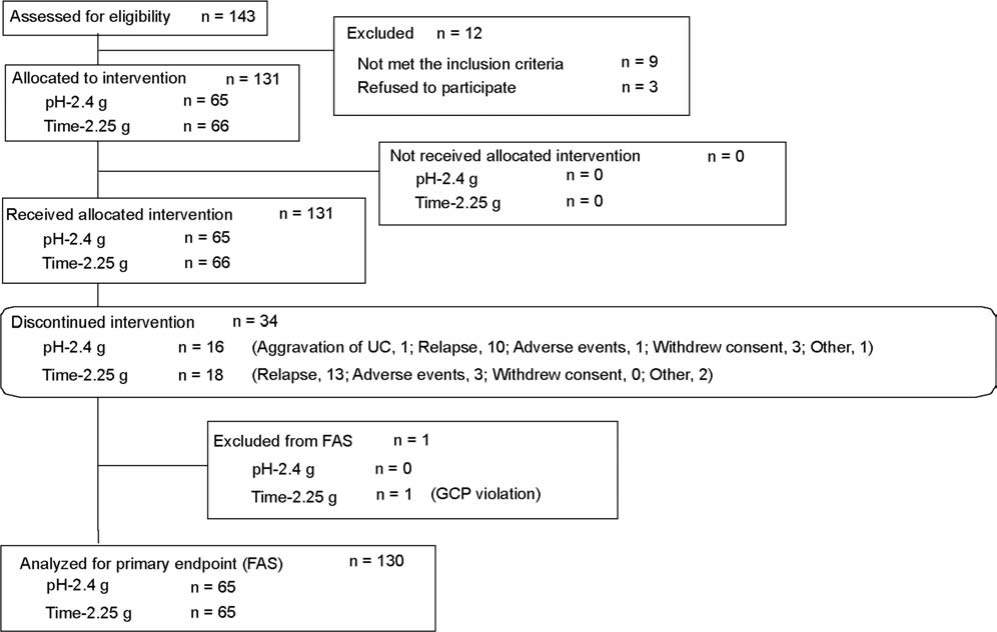
Enrolment, randomization, and follow-up of the study patients.

Investigators obtained informed consent from 143 patients during the period from January to September 2006 and completed the final follow-up in September 2007 (Fig. 1). Of the 143 patients from whom consent was obtained, a total of 131 patients were assigned to the two groups (pH-2.4 g, 65; Time-2.25 g, 66). All of the 131 patients took the drug at least once. Drug compliance was greater than 75% in every patient.

A total of 34 patients (pH-2.4 g, 16; Time-2.25 g, 18) withdrew from the study. The most frequent reason for withdrawal was relapse of UC based on the discontinuation criteria of a bloody stool score of 1 or more and UC-DAI of 3 or more (pH-2.4 g, 10; Time-2.25 g, 13), and the second most common reason was the occurrence of AEs (pH-2.4 g, 1; Time-2.25 g, 3).

There were 130 patients in the FAS (pH-2.4 g, 65; Time-2.25 g, 65) and 126 patients in the PPS (pH-2.4 g, 64; Time-2.25 g, 62). The results were very similar when the data were analyzed according to the FAS or PPS. Therefore, the result analyzed according to the FAS will be shown at the following. We did not perform adjustments for demographic factors because patient demographics in all groups were similar (Table 1).

**TABLE 1:** Patient Demographics

	pH-2.4 g (*n* = 65)	Time-2.25 g (*n* = 65)
Sex (male/female)	40/25	41/24
Age (years)		
Mean	43.4	42.6
SD	12.0	10.5
Weight (kg)		
Mean	62.01	60.15
SD	12.35	11.76
Years of disease duration (no. of patients)		
<1	5	9
<2	7	9
<3	5	7
<4	5	7
<5	2	5
≥5	41	28
Inflamed areas (no. of patients)		
Proctitis-type	23	27
Others	42	38
Years of present remission (no. of patients)		
<2	44	46
≥2	21	19

### Efficacy

The proportion of patients without bloody stools was 76.9% in pH-2.4 g and 69.2% in Time-2.25 g (Table 2). The difference between the two groups was 7.7% (95% CI: −7.4, 22.8), and the lower limit of CI was more than “−10.0%”, the critical value for demonstration of predetermined noninferiority.

**TABLE 2:** Bloody Stool, Relapse, and Decrease in the UC-DAI

	pH-2.4 g (*n* = 65)	Time-2.25 g (*n* = 65)	Difference
Bloody stools			
No. of patients	65	65	
Presence	15	20	
Absence	50	45	
Absence (%)	76.9	69.2	7.7
(95% CI)	(64.9, 86.4)	(56.6, 80.0)	(−7.4, 22.8)
Relapse			
No. of patients	65	64	
Presence	13	13	
Absence	52	51	
Absence (%)	80.0	79.7	0.3
(95% CI)	(68.3, 88.8)	(67.8, 88.7)	(−13.5, 14.1)
Decrease in UC-DAI			
No. of patients	57	59	
Mean	−0.8	−0.9	0.1
SD (95% CI)	2.4 (−1.4, −0.2)	2.3 (−1.4, −0.3)	(−0.7, 0.9)

In the comparison of the frequency of relapse, one patient was excluded because the mucosal appearance data were missing in Time-2.25 g. Decrease in the UC-DAI was calculated from the scores at the initial and final assessments. The data of 14 patients (pH-2.4 g, 8; Time-2.25 g, 6) had to be excluded from the analysis because the mucosal appearance data were missing.

**FIGURE 2:**
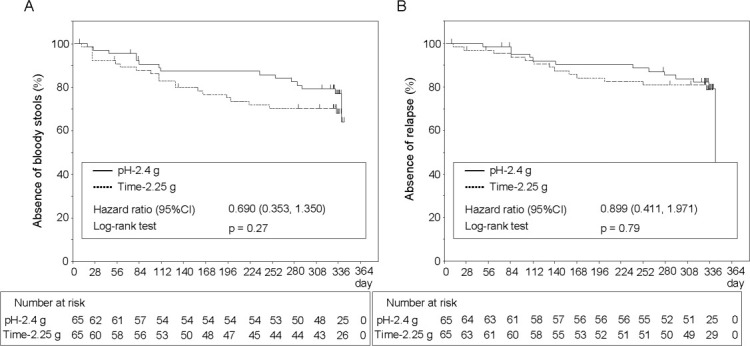
Time to bloody stools and time to relapse. The graphs show survival curves in patients without bloody stools (A) and without relapse (B). The number of patients maintained on each drug is shown below the graph.

The HR for time to bloody stools was 0.690 (95% CI: 0.353, 1.350, Fig. 2). There was no significant difference in the results of the log-rank test between the two groups (*P* = 0.27), but the time to bloody stools tended to be longer in pH-2.4 g in comparison with Time-2.25 g. The proportion of patients without relapse was 80.0% in pH-2.4 g and 79.7% in Time-2.25 g (Table 2). The time to relapse also was prolonged in pH-2.4 g compared to Time-2.25 g, in a similar manner to the time to bloody stools (Fig. 2), but the difference was not statistically significant (*P* = 0.79). The decrease in UC-DAI at the final assessment was −0.8 in pH-2.4 g and −0.9 in Time-2.25 g, respectively, and the difference between the two groups was not significant (Table 2).

Table 3 summarizes the relationship between the patients with bloody stools and the patients with relapse. In pH-2.4 g, 13 of the 15 patients with bloody stools experienced a relapse. In Time-2.25 g, 13 of the 19 patients with bloody stools experienced a relapse (one of the 20 patients with bloody stools was excluded for a missing mucosal appearance score).

**TABLE 3:** Relapse and Decrease in the UC-DAI According to Whether Patients Had Bloody Stools

		pH-2.4 g (*n* = 15)	Time-2.25 g (*n* = 20)	Difference
A				
Patients with bloody stools				
Relapse	No. of patients	15	19	
	Presence	13	13	
	Absence	2	6	
	Absence (%)	13.3	31.6	
Decrease in UC-DAI	No. of patients	10	16	
	Mean	−5.0	−3.1	−1.9
	SD (95% CI)	2.4 (−6.7, −3.3)	3.1 (−4.7, −1.5)	(−4.2, 0.4)

		pH-2.4 g (*n* = 50)	Time-2.25 g (*n* = 45)	Difference

B				
Patients without bloody stools				
Relapse	No. of patients	50	45	
	Presence	0	0	
	Absence	50	45	
	Absence (%)	100.0	100.0	
Decrease in UC-DAI	No. of patients	47	43	
	Mean	0.1	0.0	0.1
	SD (95% CI)	1.2 (−0.2, 0.4)	1.1 (−0.3, 0.3)	(−0.3, 0.5)

Decrease in UC-DAI was calculated from the scores at the initial and final assessments.

A: Comparison of the frequency of relapse and the decrease in the UC-DAI in the patients with bloody stools. In judgments of the presence or absence of relapse, the data of one patient in 20 patients with bloody stools in Time-2.25 g was excluded because the mucosal appearance data were missing. B: Comparison of the frequency of relapse and the decrease in UC-DAI at the final evaluation in the patients without bloody stools.

[correction made to table after initial online publication].

### Reliability of the Mucosal Appearance Scores

Table 4 summarizes the proportion of agreement in judgments by the image assessment committee and the evaluations by the investigators. The proportion of agreement was 69.3%, and Cohen's κ coefficient was 0.486.

**TABLE 4:** Agreement Between Evaluations by the Investigators and Judgments by the Image Assessment Committee

		Evaluations by the Investigators					
n = 114		0	1	2	3	Total	
Judgments by committee	0	41	7	0	0	48	Proportion of agreement (%) 69.3
	1	13	34	10	0	57	
	2	0	3	4	2	9	
	3	0	0	0	0	0	Cohen's κ coefficient 0.486
	Total	54	44	14	2	114	

Proportion of agreement (%) = (number of agreement cases) / (number of cases confirmed by colonoscopy) × 100

In this trial, 131 patients were allocated to an intervention. The data of 16 patients had to be excluded from the analysis because the mucosal appearance score was missing, and the data of one patient had to be excluded from the analysis because of a GCP violation.

### Safety

There were no statistically significant differences between the two groups regarding AEs and ADRs (Table 5). Serious AEs consisted of aggravation of UC in 3 patients (pH-2.4 g, 2; Time-2.25 g, 1). The investigators did not rule out a causal relationship to the drug in 1 patient in the pH-2.4 g group.

**TABLE 5:** Adverse Events and Adverse Drug Reactions

	pH-2.4 g (*n* = 65)		Time-2.25 g (*n* = 65)	
	No. of Patients	(%)	No. of Patients	(%)
Adverse events*	62	(95.4)	62	(95.4)
Nasopharyngitis	32	(49.2)	31	(47.7)
Diarrhea	4	(6.2)	9	(13.8)
C-reactive protein increased	19	(29.2)	18	(27.7)
Beta-N-acetyl-D-glucosaminidase increased	13	(20.0)	18	(27.7)
Eosinophil count increased	11	(16.9)	14	(21.5)
Blood bilirubin increased	10	(15.4)	8	(12.3)
Bilirubin conjugated increased	9	(13.8)	5	(7.7)
Alanine aminotransferase increased	7	(10.8)	6	(9.2)
Monocyte count increased	6	(9.2)	10	(15.4)
Adverse drug reactions	29	(44.6)	31	(47.7)

Events that occurred in more than 10% of the patients in at least one group.

## DISCUSSION

Previous randomized controlled studies showed that the pH- and time-dependent release formulations of mesalamine used in the present study were superior to placebo.^13^,^14^ However, the criteria for clinical relapse differed in the individual studies. Moreover, the studies employed different administration periods and doses, and such differences made it impossible to compare their results. In the present study we demonstrated the noninferiority of the pH-dependent release formulation of mesalamine to its time-dependent release formulation. The results of the present study were meaningful because this study was conducted with concurrent control.

Sutherland et al^10^ found that decreases in bloody stool score and in the mucosal appearance score paralleled symptomatic improvement. In our study, UC-DAI increased in the patients with bloody stools in both groups, whereas there was little change in UC-DAI in the patients without bloody stools in either group (Table 3). These results indicate that there is a close relation between clinical relapse (based on the clinical activity index) and bloody stools (based on the patient interviews). Therefore, we consider the presence or absence of bloody stools to be a useful predictor of clinical relapse in patients with UC in the remission phase.

Because UC is characterized by repeated relapses and remissions, the goal of treatment is to maintain the remission phase as long as possible in order to maintain the QOL of the patient.^1^,^2^ The prolongation of the remission phase is, therefore, an important indicator of the clinical efficacy of UC therapy. In the present study the time to bloody stools and the time to relapse tended to be longer in the group treated with the pH-dependent release formulation, but the difference was not significant (Fig. 2). We assume that the drug-release mechanism described previously was responsible for the improvement in symptoms seen with the pH-dependent release formulation in this study: pH-dependent release formulations might be favorable because of higher mesalamine concentrations in the mucosa. An inverse correlation between the UC-DAI and mucosal mesalamine concentration has been reported in patients without bloody stools.^15^ Accordingly, the pH-dependent release formulation may deliver an adequate amount of mesalamine to the inflamed area and effectively suppress symptom aggravation for longer periods.

Comparison of the judgments by the image assessment committee and the evaluations by the investigators revealed a consistency of ≈70% (Table 4). The Baron score is an endoscopic index that is popular in evaluating the severity of the mucosal appearance.^16^ Hirai and Matsui^17^ reported a relationship between the scores by two raters who employed the Baron score. In their study the proportion of agreement and κ coefficient between two raters were 51% and 0.31, respectively, but their coefficient was lower compared to our study. In the Hirai and Matsui study, 8.7% of all patients observed two degrees of difference in the scores between two raters. On the other hand, in our study there were no cases showing two degrees of difference. Thus, we assumed that the interobserver variation among the investigators was well controlled in our study.

There were no differences in the safety profiles between the groups (Table 5). The proportion of patients who experienced AEs was high in our study, at ≈95% in each group. This high proportion is likely attributable to the long administration and long follow-up periods. Additionally, all of the frequent events shown in Table 5 were mild, and there were no severe events causally related to either drug. Thus, no particular safety issue regarding long-term use of either mesalamine formulation was raised in this study. The finding is consistent with the current consensus that oral mesalamine is a safe and effective treatment for UC.

In summary, this is the first study to directly compare the efficacy and safety of pH- and time-dependent mesalamine formulations for the maintenance of remission in patients with UC. The results show that the pH-dependent release formulation of mesalamine evaluated in this study is as effective as the time-dependent formulation. In addition, the pH-dependent release formulation tended to prolong the duration of the remission phase. These results imply that treatment with the pH-dependent release formulation is capable of improving the QOL of patients with UC in the remission phase. However, further study is needed to clarify precisely how the drug-release mechanism contributes to the prolongation of the remission phase. Once it is clarified, it may be feasible to select the optimal mesalamine formulation for each patient in accordance with the individual patient's disease phenotype.
